# Perforator plus flap: Evolution of the concept and its place in plastic surgeons repertoire

**DOI:** 10.4103/0970-0358.73426

**Published:** 2010

**Authors:** Ramesh Kumar Sharma

**Affiliations:** Department of Plastic Surgery, Postgraduate Institute of Medical Education and Research, Chandigarh, India

The concept of ‘perforator plus flap’ was first envisaged in our unit and has evolved over the years. This nomenclature was initially coined by us and published in 2005.[1] All the surgeons of the department (including the residents) have been regularly employing this principle both in emergency and routine set up at PGI, Chandigarh. One of the authors of this paper was plastic surgery resident with us during conceptualization of this technique. The present study illustrates the application of this novel method in a variety of clinical situations.

‘The perforator plus’ technique combines the advantages of providing additional blood supply and safeguarding the venous return. The peninsular nature of the flap further prevents any kinking of the perforator vessels. Although the movement in a ‘perforator plus’ flap is less than in an island flap, this loss of mobility is compensated by better venous return and additional blood supply.

In a classical rotation design, the moving tip of the flap is under tension if the donor defect is closed primarily. This can be avoided by either planning an oversized arc of rotation or by making a back cut from the existing pivot point into the base of the flap.[2–4] In a traditional design of the flap, this back cut can compromise the blood supply. The ‘perforator plus’ peninsular flap has prior identified perforator(s) in the base and, therefore, gives freedom to make a back cut without any fear of compromising the blood supply. A back cut moves the pivot point closer to the defect thereby permitting better movement of flap and easing tension on the advancing edge. This principle is applicable to rotation, transposition, interpolation or any other design of a peninsular flap. Figure 1 illustrates the mechanism of execution of a perforator plus design in a rotation and transposition flap for a defect. These principles can be applied both to cutaneous and dermal/subcutaneous peninsular flaps. The two examples would illustrate this point.

**Figure 1:**
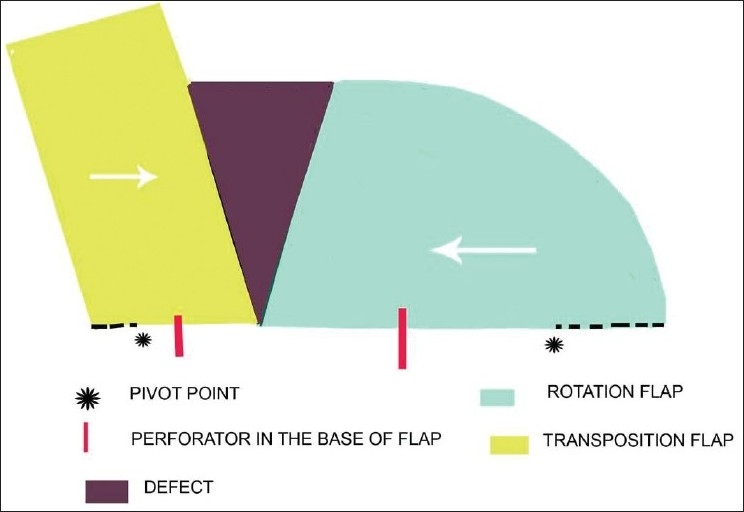
Presence of a perforator allows giving a back cut without fear of vascular compromise both in rotation (right side of defect) and transposition designs (left side of defect).

## CASE 1

**Figure 2:**
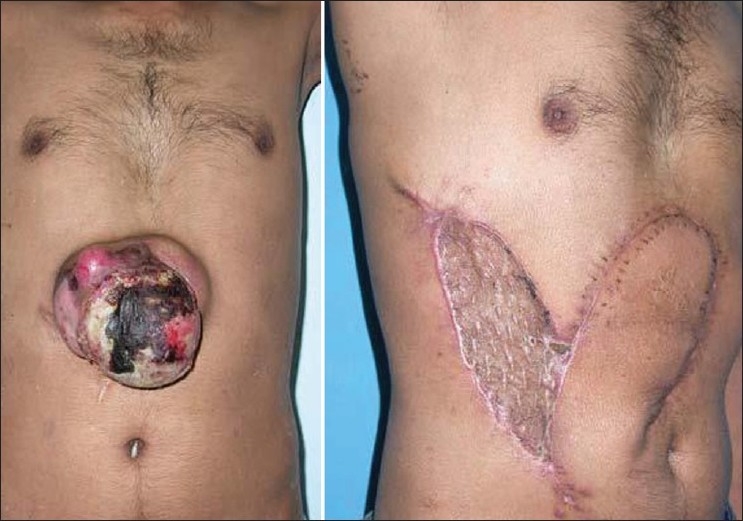
Example of a cutaneous ‘perforator plus’ flap in the thoracoumbilical region allows closure of a large defect.

This patient with abdominal wall tumour had a full thickness defect after tumour extirpation that necessitated a flap cover. A local flap incorporating periumbilical perforators of deep inferior epigastric artery was planned. The base of the flap was narrowed down by giving a back cut till the flap could easily be transferred into the defect. The flap survived completely and patient had an uneventful recovery [Figure 2].

## CASE 2

**Figure 3:**
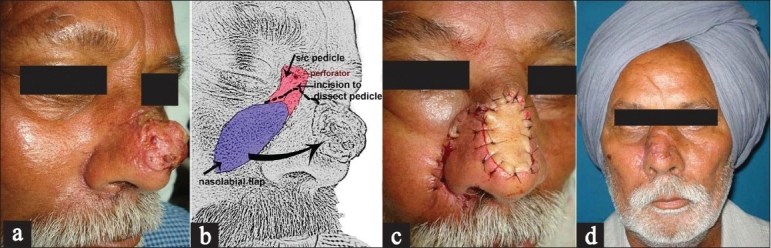
Example of a dermo-subcutaneous-fat ‘perforator plus’ flap. (a) Squamous cell carcinoma nose. (b) Dermo-subcutaneous fat pedicle nasolabial flap was designed that incorporated the previously mapped perforator. (c) Transferred flap. (d) Well-healed flap at 3 months

Excision of squamous cell carcinoma over the dorsum of nose resulted in exposure of underlying bones and cartilages necessating a flap cover in this patient. It was decided to interpolate a nasolabial flap incorporating a perforator from the angular vessel. A dermo-subcutaneous fat pedicle flap was designed that incorporated the previously mapped perforator [Figure 3 a and b]. The flap was successfully transferred into the defect [Figure 3c]. The flap healed well and in [Figure 3d] we can see the 3 months postoperative appearance.

This technique indeed is a very useful tool in the armamentarium of the plastic surgeon. We have found it to be particularly handy in emergency situations involving trauma to limbs, trunk or head and neck. Majority of the flaps are raised by our residents in emergency and have done very well in most of the instances. We shall recommend more liberal and frequent use of this method in day to day practice.
